# Nanotechnology-based non-viral vectors for gene delivery in cardiovascular diseases

**DOI:** 10.3389/fbioe.2024.1349077

**Published:** 2024-01-18

**Authors:** Liping Jiao, Zhuokai Sun, Zhihong Sun, Jie Liu, Guanjun Deng, Xiaozhong Wang

**Affiliations:** ^1^ The Affiliated Yantai Yuhuangding Hospital of Qingdao University, Yantai, China; ^2^ Queen Mary School, Nanchang University, Nanchang, China; ^3^ School of Pharmaceutical Sciences (Shenzhen), Sun Yat-Sen University, Shenzhen, China; ^4^ The Second Affiliated Hospital of Nanchang University, Nanchang, China; ^5^ School of Public Health, Nanchang University, Nanchang, China

**Keywords:** non-viral vector, gene therapy, cardiovascular disease, gene transfer, nanotechnology

## Abstract

Gene therapy is a technique that rectifies defective or abnormal genes by introducing exogenous genes into target cells to cure the disease. Although gene therapy has gained some accomplishment for the diagnosis and therapy of inherited or acquired cardiovascular diseases, how to efficiently and specifically deliver targeted genes to the lesion sites without being cleared by the blood system remains challenging. Based on nanotechnology development, the non-viral vectors provide a promising strategy for overcoming the difficulties in gene therapy. At present, according to the physicochemical properties, nanotechnology-based non-viral vectors include polymers, liposomes, lipid nanoparticles, and inorganic nanoparticles. Non-viral vectors have an advantage in safety, efficiency, and easy production, possessing potential clinical application value when compared with viral vectors. Therefore, we summarized recent research progress of gene therapy for cardiovascular diseases based on commonly used non-viral vectors, hopefully providing guidance and orientation for future relevant research.

## 1 Introduction

Cardiovascular disease (CVD) leads to almost a third of all deaths worldwide, resulting from atherosclerotic plaque leading to hemadostenosis and blood flow restriction (Park et al., 2020; Tsao et al., 2022). Despite progress in medical technology, CVD is still a major cause of death (Yang et al., 2023). Conventional treatment strategies for CVD include anticoagulation, antiplatelet, thrombolytics, hypolipidemic drugs, and invasive therapies like vascular bypass grafting and stent transplantation (Zhu et al., 2021). However, small molecule drug therapy in conventional treatment strategies is characterized by short half-life and low bioavailability, and long-term use of certain drugs may also lead to side effects such as drug resistance and potential hematological toxicity (Missri, 1979; Fu et al., 2014). Surgical treatment, on the other hand, is more pro-traumatic, requires a longer recovery time, and has a high risk of postoperative complications. These problems have led to the fact that conventional treatment options cannot fully meet clinical needs (Harafuji et al., 2005; Pala et al., 2020). In addition, conventional therapy focuses on palliatives to manage symptoms and slow down the disease progression, rather than disease eradication (Markina et al., 2023).

Gene therapy is a technique that rectifies defective or abnormal genes by introducing exogenous genes into target cells to cure the disease (Korpela et al., 2021). Delivering genes into targeted cells and tissues through vectors is crucial in gene therapy (Jiao et al., 2020). On the premise of the inactivation of the host immune response, transferring therapeutic genes into host cells with efficiency and sustainability is key to the success of gene therapy (Yetisgin et al., 2020). Virus and plasmid DNA are the most ordinary vectors in gene therapy agents (Hou et al., 2022). In practice, however, only a little bare plasmid DNA enters the cells, resulting in low transfection efficiency of therapeutic genes in target tissues (Ylä-Herttuala and Martin, 2000). Only adenovirus (Ad) vectors and adenovirus associated (AAV) vectors have been applied in cardiac clinical trials (Yetisgin et al., 2020), but potential risks like integration with the host cell genome and nonnegligible immunogenicity exist (Kaski and Consuegra-Sanchez, 2013; Yin et al., 2014; Lin and Qi, 2023). Non-viral vectors can effectively compensate for the shortcomings of viral vectors and safely achieve effective gene therapy (Expósito et al., 2023). As nanotechnology advances continue, gene therapy based on nanocarriers has entered clinical trials due to its unique properties and advantages.

**FIGURE 1:**
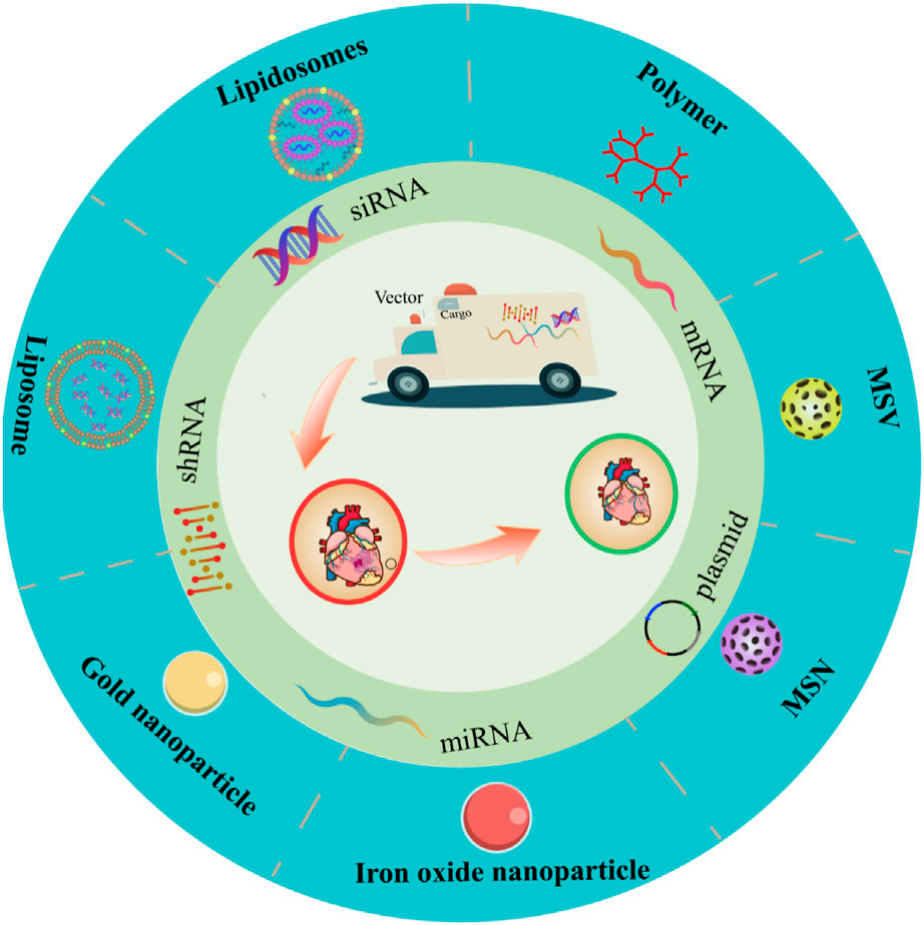
Types of non-viral vectors for gene therapy in cardiovascular diseases.

Nanotechnology-mediated non-viral vector is a gene delivery system utilizing non-viral nanomaterials, as a safer alternative to viral vectors due to high safety and low cost (Sainz-Ramos et al., 2021). Non-viral nanocarriers generally include polymers, liposomes, lipid carriers and inorganic materials (Gu et al., 2020). Non-viral nanocarriers take advantage of physicochemical properties to modify vector structure (Yagublu et al., 2022), transferring exogenous nucleic acids to the targeted cells and assisting them to escape from the endosomes to ensure efficient expression (Yang et al., 2022). In comparison with viral vectors, non-viral nanocarriers, with higher biosafety, lower immunogenicity, and mutagenicity, own a wider application owing to convenient process, low cost, and no restriction on the target gene size (Yin et al., 2014; Ren et al., 2021). Recently, much progress has been achieved in nanotechnology-mediated gene therapy in the treatment of CVD as an intensive pathogenesis study and the development of personalized precision medicine. Based on this, this review summarized the latest researches on non-viral nanocarriers of gene therapy for CVD (Figure 1).

## 2 Application of non-viral vectors in cardiovascular diseases

### 2.1 Polymer nanoparticles

Polymer-based nanocarriers are fabricated from natural or synthetic polymers in terms of component variety and structure diversity (Mendes et al., 2022). As one of the most promising materials for nucleic acid nanodelivery, polymer nanocarriers have significant advantages in synthesis and functional modification, transfection efficiency, biocompatibility, etc. (Piotrowski-Daspit and Alexandra, 2020). For gene delivery, polymer nanocarriers mainly condense and encase nucleic acid through the electrostatic interaction between the cationic primary amine (cationic polymer) and the anionic phosphate group (nucleotide) without being degraded or cleared by enzymes in the reticuloendothelial system (Rupei et al., 2011; Ho et al., 2021). Furthermore, polymer nanocarriers notably enhance gene transfection efficiency by interrupting and escaping from endosomes through the “proton sponge” effect and high buffering ability within the physiological pH (Yan et al., 2022). Although they have excellent transfection efficiency, conventional cationic polymers are limited in clinical application because of non-degradability, cytotoxicity, aggregation, and lack of cell specificity (Askarian Saeedeh et al., 2015; Hao et al., 2019). Experiments aimed at polymer modification, transformation, or innovation have been conducted to overcome these deficiencies. For instance, by introducing hydrophilic polymers (typical PEG chains), serum stability and biocompatibility have been considerably improved (Mendes et al., 2017; Mendes et al., 2022). In this way, targeting moieties (antibodies, transferrin, folic acid, and glycosyl components) were attached to the surface of the polymer and then the cell uptake and target specificity were facilitated via targeted gene-molecule mutual interaction (Mitchell et al., 2021).

**FIGURE 2:**
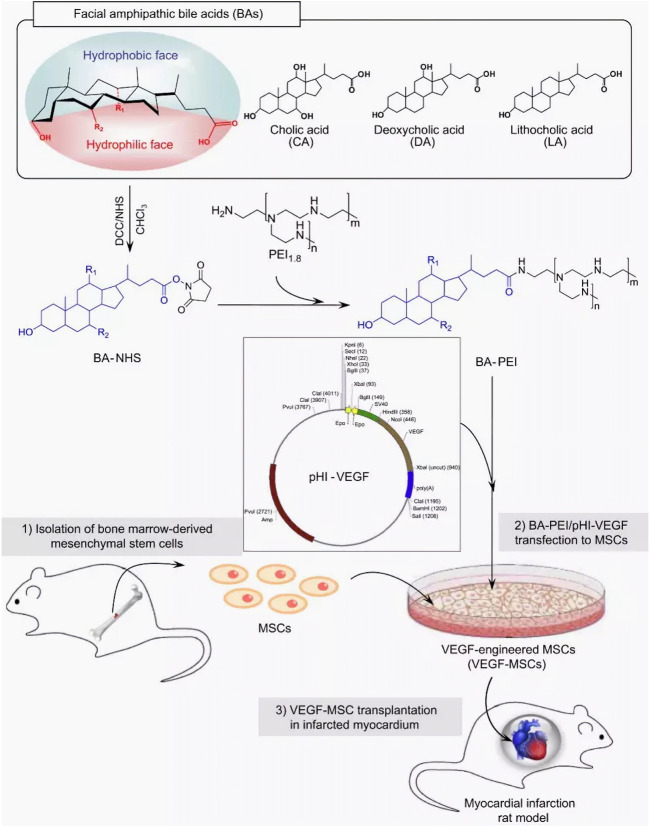
Schematic representation of the synthesis of BA-PEI conjugates and VEGF-MSC transplantation in infarcted myocardium. BA-PEI/pHI-VEGF was used to transfect VEGF into rat MSCs to overexpress VEGF protein and play a therapeutic role in the rat myocardial ischemia-reperfusion model (Moon et al., 2014).

Boussif et al. synthesized a linear and branched polycationic polymer -polyethyleneimine (PEI) (Boussif, 1995), which has been used as a “gold standard” for gene delivery of non-viral vectors (Mitchell et al., 2021; Tong et al., 2023). Modification and functionalization of PEI have optimized efficiency and safety in gene delivery, making it widely used in CVD gene therapy. For example, bile acid-modified PEI (BA-PEI) with an amphiphilic surface was designed by (Moon et al., 2014) (Figure 2). BA-PEI successfully delivered hypoxia-induced VEGF (pHI-VEGF) into MSCs via increasing plasma membrane permeability, which enabled VEGF overexpression under ischemia and thus alleviated left ventricular remodeling after acute myocardial infarction (AMI). Wang et al. found that the positively charged hyperbranched PEI could be transformed into a non-toxic polymer material with hydrazide by neutralizing the primary amine group of dendritic macromolecules with the peripheral hydrazide group. With further modification of polypeptide ligands, a new PEI-based carrier (PEI-HYD-RGD) was created, which showed good biocompatibility and cell internalization efficiency when equipped with siRNA (PEI-HYd-RGD-SIRNA) *in vitro* (Wang et al., 2016). In the zebrafish cardiac injury model, PEI-HYd-RGD-SIRNA could be significantly absorbed by cardiomyocytes and endothelial cells in the injured ventricular apex region. This innovative PEI-based vector is expected to be further developed into a siRNA therapy agent for cardiovascular disease. Compared to PEI alone, Yu et al. (Yu et al., 2021) constructed a miRNA delivery system based on silica and PEI (F-silica Mir-24), which could release the loaded miRNAs (Mir-24) into the cytoplasm to inhibit apoptosis. Overexpression of miR-24 inhibited cardiomyocyte apoptosis and fibrosis by directly targeting and suppressing the expression of the pro-apoptotic protein Bim. This strategy enhanced new function and long-term prognosis 7–10 days after acute myocardial infarction, providing a valuable approach to the treatment of acute myocardial infarction.

Polyamidoamine (PAMAM) is one of the earliest carriers used for gene delivery and one of the most commonly used dendrimeric cationic polymer carriers in biological applications (Zu and Gao, 2021). By modifying its structure and introducing antibodies on its surface, its delivery efficiency in CVD can be improved. For example, K. Zhu et al. (Zhu et al., 2013) synthesized hyperbranched PAMAM (h-PAMAM) as a novel gene delivery vector through polycondensation. The pHRE-Hvegf165 carried by h-PAMAM can resist nuclease digestion and efficiently transfect primary skeletal myoblasts (SKMs) with low cytotoxicity. In the myocardial infarction model, the transfected SkMs can reduce infarct area and interstitial fibrosis, thus inhibiting left ventricular remodeling in the post-infarction period and enhancing cardiac function. Furthermore, another study cross-linked the PAMAM-DNA complex with anti-e-selectin mab (1.2B6), delivering the PAMAM-DNA complex to the inflammatory vascular endothelium by binding the 1.2B6 antibody to E-selectin and P-selectin. As a result, the delivered anti-inflammatory genes reversed or inhibited atherosclerosis progression (Theoharis et al., 2009).

Poly (lactic-co-glycolic acid) (PLGA), one of the most successful synthetic biodegradable polymers, is hydrolyzed intracellularly to lactic acid and glycolic acid that can be metabolized by the body through the Krebs cycle, and therefore has the best biocompatibility (Kim et al., 2019). PLGAs are popular as carriers for proteins and small molecules and are now being used to deliver genetic information to cells for gene therapy. However, PLGA polymer nanoparticles, upon entering the organism, need to cross a variety of biological barriers, such as reticuloendothelial system (RES)-mediated clearance and binding of regulatory proteins in the bloodstream to PLGA that may lead to phagocytosis and clearance of nanoparticles by macrophages (Zou et al., 2009). The presence of these barriers may limit drug delivery and absorption, thus affecting drug efficacy. To overcome these barriers, several strategies have been employed to improve the effectiveness of PLGA delivery *in vivo*. One common strategy is to provide a hydrophilic layer on the PLGA surface to encapsulate molecules that hide the hydrophobicity, and the most commonly employed hydrophilic polymer is PEG (Danhier et al., 2012). In addition, cellular uptake can be facilitated by the addition of cationic polymers (Kim et al., 2005). X. Zhang et al. (Zhang, 2022a) prepared PP/PEI nano complexes by coupling poly (ethylene glycol) methyl ether block-PLGA (PEG-b-PLGA; PP) nanoparticle surfaces with PEI, and PP/PEI would carry the CRISPR/Cas9 gene editing plasmid induced efficient genome editing in endothelial cells of the vascular system (including lungs, heart, aorta, and peripheral vasculature) of adult mice after intravenous injection. In addition, the gene editing plasmid can induce genome editing of at least two genes at the same time or introduce both genome editing and transgene expression in vascular endothelial cells, advancing the development of cardiovascular research and potential gene therapy. Another strategy is to attach targeting motifs to increase selective cell binding and internalization through receptor-mediated endocytosis. Messerschmidt et al. (Messerschmidt et al., 2022) Coupled anti-Tie2+Tie1 antibody to the surface of PLGA and successfully delivered the plasmid encoding Notch intracellular domain (NICD) to the zebrafish endocardial layer via antibody binding to the endocardium, which resulted in the overexpression of Notch-related genes and significant improvement in cardiac function; T. Wang et al. (T. Wang et al., 2022) used platelet vesicle (PMV) camouflaged PLGA nanoparticles (PMVs@PLGA) as a carrier for miRNA inhibitors, and miRNA-targeted transport into cardiomyocytes indirectly increased the expression of Nuclear factor (erythroid-derived 2)-related factor 2 (Nrf2) by competitively binding to miR-155-5p, which protectsed myocardium in the occurrence of myocardial ischemia-reperfusion injury (MIRI), and provided a new potential pathway for the targeted treatment of MIRI.

Chitosan (CS) as a common natural carbohydrate polymers (Zu and Gao, 2021), is an alkaline polysaccharide with high biodegradability, biocompatibility, and nontoxicity and a 6.5 apparent pKa. Most of the amino groups are protonated under acidic conditions (Chronopoulou et al., 2022). Therefore, chitosan is an ideal carrier for delivering nucleic acids due to its ability of nucleic acids absorbance to form stable complexes (Ashrafizadeh, 2023). Besides, CS surface modification by PEG with different molecular weights improved aqueous solubility and prolonged half-lives (Zhang et al., 2018). Nguyen et al. used sodium tripolyphosphate (TPP) (a crosslinking agent) to synthesize polyethylene glycol chitosan polymer (chNPs) through an ester coupling reaction to enclose miR-33, and then successfully formulated an intracellular gene delivery vector targeting mouse macrophages (Nguyen et al., 2019). The results showed that chNPs successfully inhibited ABCA1 expression thereby reducing the outflow of liposterols in cholesterol metabolism, which could be used to treat atherosclerosis. Zhou et al. (Zhou et al., 2016) modified trimethyl chitosan (TMC) with the short peptide REDV (Arg-Glu-Asp-Val) and PEG to deliver miR 126 into vascular endothelial cells (VECs), which has achieved remarkable progress in promoting VECs proliferation and improving ischemic myocardial necrosis due to the high transfection efficiency.

### 2.2 Liposome nanoparticles

Liposomes are closed, spherical vesicles that consist of a phospholipid bilayer with polar head groups and non-polar tail groups, along with a stabilizer like cholesterol. These liposomes are capable of delivering drugs such as nucleic acids into the cells (Mukalel et al., 2019; Li et al., 2019). Cationic liposomes are a general term for a class of positively charged liposomes, which are typically composed of diverse cationic lipid molecules alone or with neutral auxiliary lipids, such as 1,2-dioleoyl-SN-propyltriphenyl (3) phosphatidyl ethanolamine (DOPE), phosphatidylcholine (PC), cholesterol (Chol/CHO), and other common composition (Ma et al., 2021). Cationic liposomes play a crucial role in cationic liposomes by providing a positive charge, which enables them to attract, encapsulate, and compress nucleic acids (Montoto et al., 2020), while the helper lipids are in charge of improving bilayer membrane stability, reducing cationic liposome toxicity, and promoting their ability to escape from endosomes (Imani et al., 2018; Buck et al., 2019). The positively charged carrier-nucleic acid complex, through the action of electrostatic interaction, is adsorbed to the cell surface and enters the cell via endocytosis and membrane fusion, ultimately achieving transgenic expression (Shtykalova et al., 2023).

Liposomes have many merits as gene delivery vectors. Firstly, liposomes are spherical vesicles that encapsulate nucleic acids and resist nucleases (Hussen and Mahmud, 2023). Secondly, similar to cell membranes, liposomes are easy to fuse with recipient cells and have high transfection efficiency. Thirdly, as delivery systems, liposomes have no limits in the host (Lim et al., 2020). Finally, the phospholipid bilayer structure highly mimics cell membranes and exhibits excellent storage stability (Li, 2022a). Although liposomes have many advantages compared with other carriers and are widely used in in vitro cell experiments and basic experimental studies, the transfection efficiency of liposomes is low in tissues or organs with special structures and arrangements such as the heart (Wang et al., 2011). In recent years, scientists have worked to solve these difficult problems by exploring and researching new methods and techniques. Among them, the targeted delivery of drugs can be improved by using nanotechnology, and the absorption rate of drugs in heart cells can be increased by rationally designed carriers (Zhao et al., 2023). Hein et al. (Hein et al., 1998) used transferrin-modified cationic liposomes to deliver plasmids expressing vascular endothelial growth factor (pCMVbeta) into vascular endothelial cells. This approach significantly improved the delivery efficiency of the plasmid compared to unmodified vectors.

**FIGURE 3:**
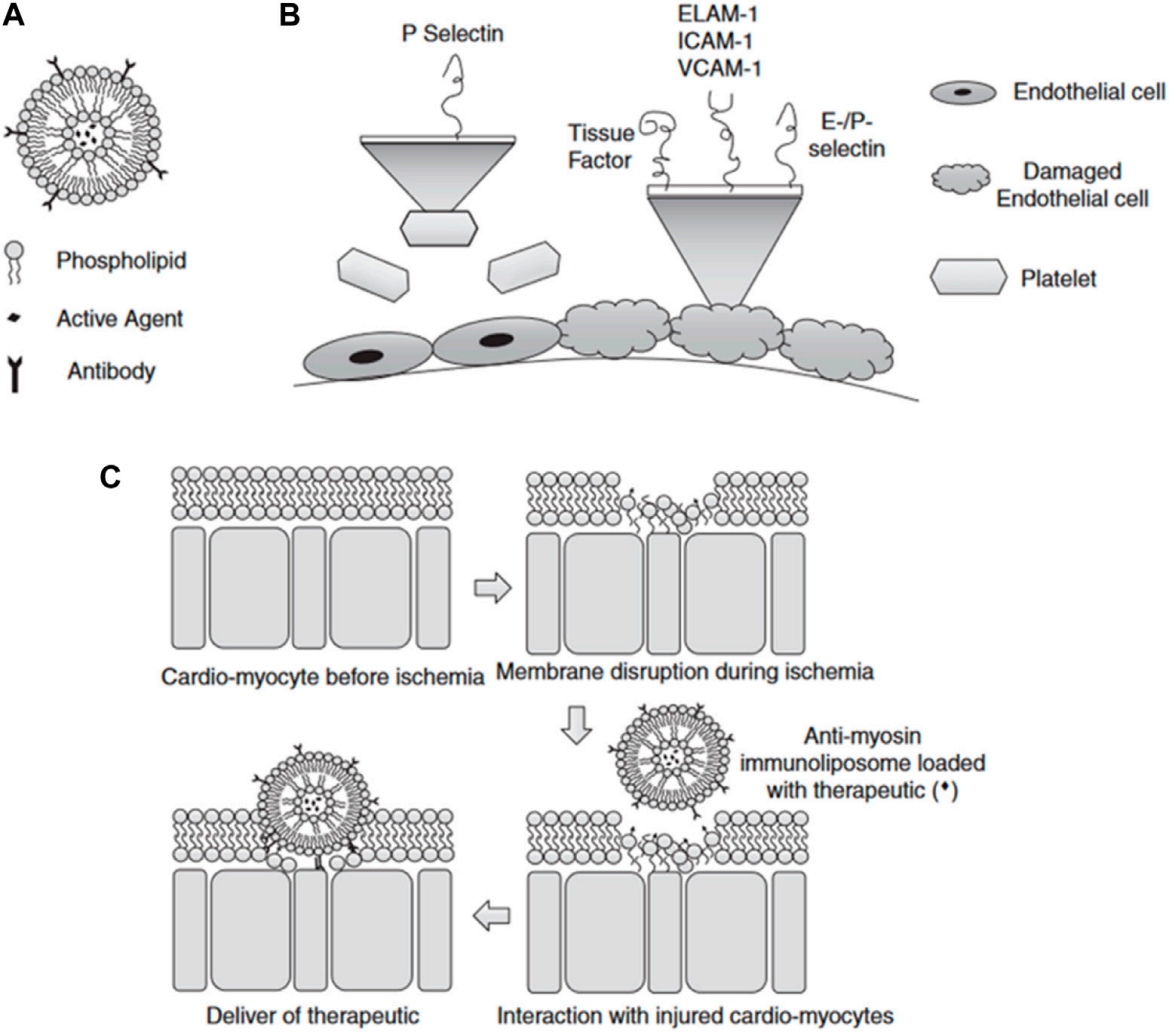
**(A)** Schematic diagram of liposomes **(B)** Selection of targets when endothelial damage occurs **(C)** Schematic diagram of the mechanism of liposomes targeting ischemic cardiomyocytes (Bowey et al., 2012).

Cardiovascular diseases, such as atherosclerosis, restenosis, and inflammation, often occur in specific areas of the vasculature, offering the potential for targeted drug therapy applications. Liposomes are promising targeted drug carriers for intravascular applications that could beneficially impact the treatment of these conditions (Tang et al., 2021; Lestini et al., 2002). Liposomes utilize both passive and active targeting approaches to enhance the delivery efficiency and residence time of payload genes in the body (Li Wenpan et al., 2022). Liposome size, charge, and surface modification can significantly impact their blood clearance, cellular uptake, and distribution throughout the cardiovascular system (Khadka et al., 2020; Manners et al., 2022). Larger liposomes are prone to be engulfed by macrophages, whereas smaller ones tend to be ingested by fibroblasts (Bozzer, 2022). By modifying polyethylene glycol (PEG) on the surface of the liposome, it is disguised as an “invisible liposome” to reduce complement activation, enhance stability, and extend the cycle time of the liposome (Li et al., 2021; Zhu et al., 2017; Haghiralsadat et al., 2018). Researchers realized active targeting by adding targeting groups that improve the retention of liposomes at specific sites, increasing local concentrations and cell internalization (Bowey et al., 2012; Zhai et al., 2022). During the development of atherosclerotic lesions and injuries, the endothelium is the main location of cell extravasation and inflammation (Song et al., 2022). Leukocyte adhesion molecules (VCAM-1), ELAM-1 or E-selectin, and intercellular adhesion molecule-1 (ICAM-1) are highly expressed on the surface of vascular endothelial cells, which serve as prime targets for therapeutic delivery of CVD (Cao et al., 2021) (Figure 3). Jia et al. (X. Jia et al., 2022) designed VCAM-1 binding peptide targeting cationic liposomes (PCLs) as a siRNA delivery vector to wrap methylated NLRP3 siRNA into PCLs (NLRP3 SIRNA-PCLS). NLRP3 siRNA-PCLs can target VCAM-1 expressing endothelial cells, knockdown NLRP3, prevent TNF-α-induced NLRP3 inflammasome activation and intracellular flow of LDL, and significantly reduce the accumulation of atherosclerotic LDL in TNF-α-stimulated rat carotid endothelial cells. Another common cardiac target is myosin, which is exposed when the endothelial cell membrane is damaged. The highly specific anti-myosin monoclonal antibody 2G4 (mAb 2G4) has displayed remarkable ability in recognition and binding to ischemic cells and damaged plasma membranes, allowing intracellular myosin to be exposed to the extracellular space (Skourtis et al., 2020). Activated transcriptional activator (TAT) peptide and anti-myosin monoclonal antibody 2G4 (mAb 2G4). This construct was designed to specifically target myocardial myosin, with the aim of delivering target gene therapy to ischemic myocardium. A dual-targeted delivery system is capable of accumulating outside the cell and penetrating inside the cell to enhance the delivery of genes to target cells. Despite the many advantages of liposomes as nucleic acid carriers, the production process of preparation and nucleic acid encapsulation requires the use of organic solvent injection, which makes the production process complicated and limits the large-scale production of liposomes by their limited physical stability, low drug loading capacity, and the possibility of drug leakage (Mendes et al., 2022; Seo et al., 2023).

### 2.3 Lipid nanoparticles

**FIGURE 4:**
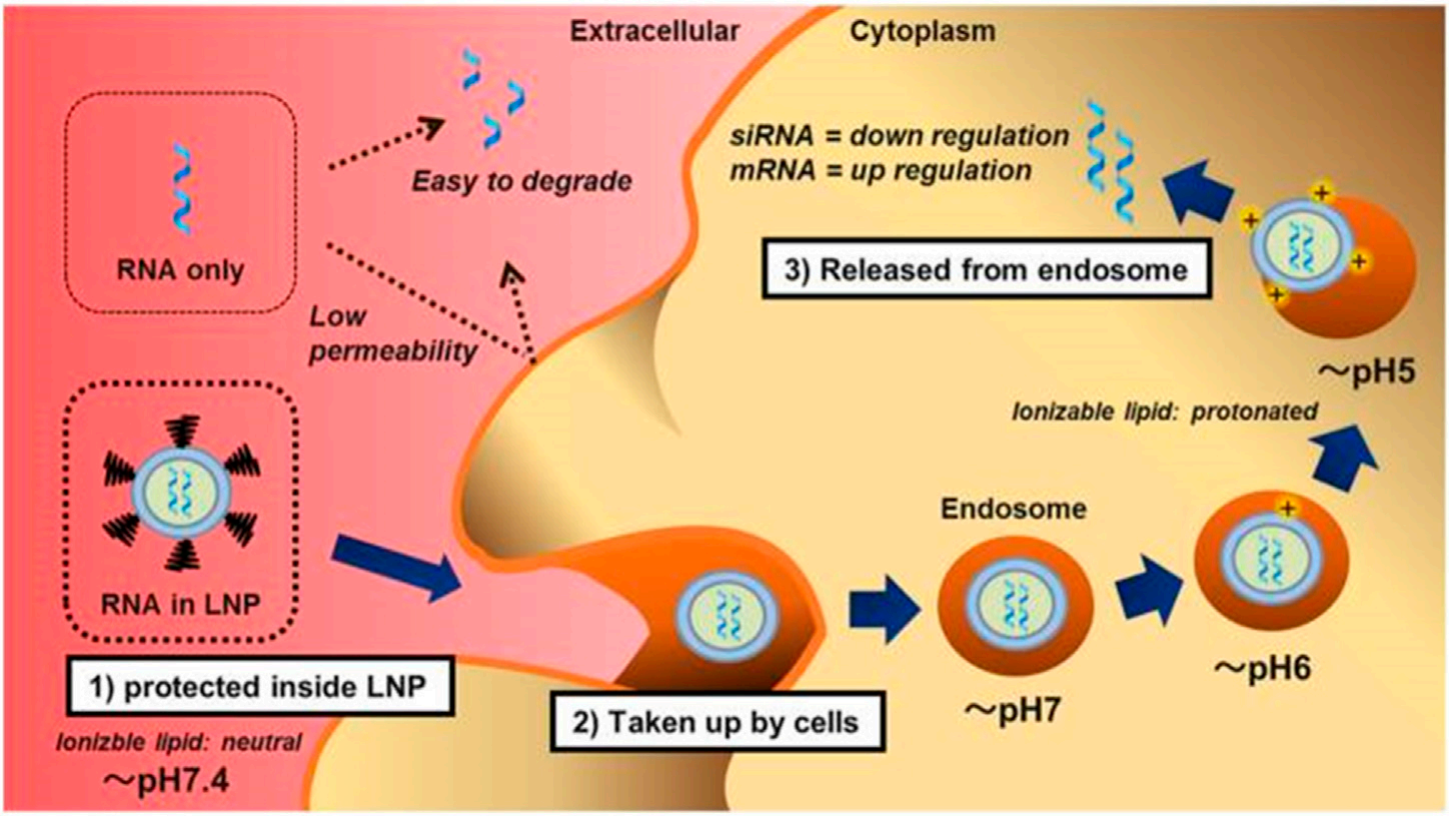
The Delivery mechanism of lipid nanoparticles (Suzuki and Ishihara, 2021). First, the lipid nanoparticles (LNP) completely encapsulated the RNA and prevented it from nuclease digestion. LNPs are neutral in physiological pH due to ionizable lipids and pegylated phospholipids, thus reducing non-specific interactions with serum proteins. Second, LNPs are taken up by cells via apolipoprotein E (ApoE) dependent and/or ApoE independent pathways when the pegylated phospholipids are dissociated. Finally, the protonated LNPs, after acidification in the endosomes, induce the hexagonal phase structure, destroy the cell membrane, and release RNA molecules into the cytoplasm.

Lipids-based nanoparticles (LNPs) are unique nanostructures, composed of various types of lipids, including cationic or ionizable lipids (CIL), structural lipids (phospholipids and cholesterol), and PEG-binding lipids (PEG-Derived phospholipids) (Patel et al., 2019). Among them, ionizable lipids are the key components that determine the titer, mRNA delivery efficiency, degradability, and reactivity (Drescher and van Hoogevest, 2020). At low pH, ionizable lipids are protonated in acidic buffers, producing a positive charge and binding to negatively charged mRNA, whereas they remain neutral at physiological pH (Chan et al., 2021; Scalzo et al., 2022). The delivery mechanism of LNPs is shown in Figure 4. Ionizable lipids are less cytotoxic than cationic lipids. This is because the positive charge of cationic lipids typically interacts with negatively charged molecules on the cell surface, leading to cell membrane damage and cell death. In contrast, ionizable lipids can dynamically regulate their charge state in the cellular environment, avoiding the continuous release of positive charges to the cell surface and reducing cytotoxicity (Sharma et al., 2014). In general, PEG-bound lipids in LNPs occupy the smallest molar percentage, which can enhance particle stability by affecting charge distribution, size, and dispersion, and hinder LNPs from aggregating, playing a crucial role in blood circulation and biological distribution (Albertsen and Camilla et al., 2022; Kon et al., 2022; Guéguen et al., 2023). Structural lipids are chiefly used to support the structure of particles and enhance stability during storage and circulation (Kulkarni et al., 2019; Witzigmann et al., 2020). According to diverse target tissues, the proportions of these four lipid components can be adjusted to change the LNP constitution (Yihunie et al., 2023). At the same time, the physical characteristics of LNPs, like particle size, morphology, encapsulation rate, and surface charge are prone to be regulated, thus producing a variety of different LNP formulations (Yang et al., 2022; Suzuki and Ishihara, 2021). Ionizable lipid nanoparticles display excellent biocompatibility, high nucleic acid encapsulation efficiency, and efficient transfection performance, releasing only under specific circumstances with low off-target effects (Yang et al., 2022). Nowadays, LNPs have become the most widely explored and applied non-viral nucleic acid delivery vector (Sun and Lu, 2023).

Now the focus is on how to improve the delivery effect of LNPs. Modifying the surface of LNPs with targeted molecular entities becomes the most popular way (Subhan et al., 2023). In principle, peptides, antibodies, or proteins that target specific cell surface molecules are added to nanoparticles carrying mRNA to block liver accumulation and enable particular mRNA transport to target cells through high-affinity binding (Kedmi et al., 2018; Yang et al., 2022). Joel G. Rurik et al. (Rurik et al., 2022) conjured CD5 antibodies to LNP (CD5/LNP) to deliver CAR mRNA encoding fibroblast activation protein (FAP). LNP vectors specifically target T cells to generate FAP- (CAR) -T cells that recognize and attack activated cardiac fibroblasts, consequently alleviating fibrosis and regaining normal heart function in a mouse model of heart failure. Studies have shown that mannose-modified LNPs (LNP-MAN) can possibly promote uptake by APCs (Zhuang et al., 2020). Gao et al. (Gao et al., 2023) self-assembled cationic lipids (G0-C14) and poly (lactide-co-ethylacetide) -B-polyethylene glycol (PLGA-PEG) that are biodegradable to form nanoparticles (HNPs), and modified mannose on HNPs by covalent bonding, formulating macrophage-targeting nanoparticles (M-HNPs) in atherosclerotic lesions. M-HNPs coated interleukin-10 (IL-10) mRNA, mediated by mannose receptor (CD206), targeted IL-10 mRNA delivery to the site of atherosclerotic lesions and translated into anti-inflammatory factor IL-10, which increased the thickness of fiber cap by decreasing the accumulation of lipids and the size of necrotic areas, thus promoting inflammation regression, inhibiting oxidative stress and apoptosis and played an anti-atherosclerosis role. Although LNP is one of the most efficient carriers for mRNA delivery, the structural differences between DNA and mRNA result in significant differences in their delivery requirements. Further optimization of LNP formulations is required to improve DNA delivery (Algarni, 2022; Li, 2022a). Scalzo et al.‘s (Scalzo et al., 2022) research team successfully induced up to 80% transfection efficiency in cardiomyocytes *in vitro* by adjusting the molar ratio of ionizable lipid C12-100 to lipid combinations and the ratio of C12-100 to plasmid DNA. This study provided a new delivery vehicle for DNA delivery in cardiomyocytes. In general, the basic structure of both LNPs and liposomes are lipid molecules with similarities, yet there are significant differences (Table 1).

**TABLE 1:** The comparison between LNP and liposome.

	LNP	Liposome
Similarities	Particle size distribution, shape, lipid composition, positive charge	
	a. Phospholipid monolayer structure	a. Phospholipid bilayer structure
	b. Reverse micelles	b. Aqueous core
Differences	c. Ethanol Dilution Method, Manual Mixing Method, T-Mix Method, Microfluidic preparation method	c. Film dispersion method, Solvent injection method, Freeze-drying method, pH gradient method
	d. The LNP-mRNA is unstable, and the storage and transportation costs are high	d. It has excellent long-term storage stability

### 2.4 Inorganic nanoparticles

Gold nanoparticles, magnetic nanoparticles, and porous silicon nanoparticles are the most widely utilized inorganic nanoparticles (Sharma et al., 2021), with precise control over shape and size, non-immunogenicity, favorable biosafety, and targeting, and are apt to large-scale production, and are extensively applied in the delivery and imaging of various drugs (Wang et al., 2023; Hu et al., 2022).

Gold nanoparticles (AuNPs) are the most widely studied metal nanoparticles with excellent physical and chemical properties (Zhang, 2022b), such as bioinert, low cytotoxicity, and high stability, and AuNPs are easy to prepare and modify, which is suitable for the delivery of nucleic acid in gene therapy (Tang et al., 2022; Lee et al., 2023). AuNPs-based application in combination with existing gene therapy has been recognized as an innovative tactic with potent possibility in treating heart disease (Pala et al., 2020). Jia et al. (Jia et al., 2017) successfully constructed Antago-Mir-155-AUNps nanoparticles by covalently binding the sulfcap-modified antago-miR-155 with AuNPs. Mice received an injection of antagomir-155-aunps via the tail vein with estrogen-deficiency induced diabetic cardiomyopathy, and antago-miR-155 was steadily released *in vivo* and predominantly sent to macrophages through phagocytosis. Antago-miR-155 mediated an increase in M2-type macrophages, reduced inflammation and apoptosis, and restored cardiac function. Therapeutic neovascularization can be completed by delivering numerous growth factors (such as VEGF) (Kim et al., 2011; Shen et al., 2021). In clinical trials, due to inferior targeting the short circulating half-life of VEGF, and the relatively long time VEGF needs to exist to prevent the degeneration of newly formed blood vessels, it is difficult for traditional intravenous VEGF administration methods to target VEGF to damaged tissues (Kim et al., 2011; Henry et al., 2003). In the mouse model of hindlimb ischemia, AuNPs, as an excellent payload, targeted exogenous VEGF to ischemic muscle tissue via its augmented permeability and retention effect, accelerated the recovery of abundant ischemic tissue and facilitated angiogenesis (Pala et al., 2020; Kim et al., 2011).

Magnetic iron oxide nanoparticles (MNP) consisting of magnetite (Fe_3_O_4_) or magnehematite (Fe_2_O_3_) have certain superparamagnetism, and magnetic liposomes (MLs) are synthesized using magnetic nanoparticles and liposomes (Arias, 2018), which can be applied in magnetic resonance imaging and magnetism-based targeted drug delivery in CVDs (Younis et al., 2021). Molavi et al. (Marcos-Campos et al., 2011) found that angiotensin receptor 1 is overexpressed in infarcted hearts and can be used as a target for MLs. Which can effectively target growth factors, biomolecules, and cytokines in the infarcted heart muscle, minimizing the affected area of cardiac fibrosis (Namdari et al., 2017).

**FIGURE 5:**
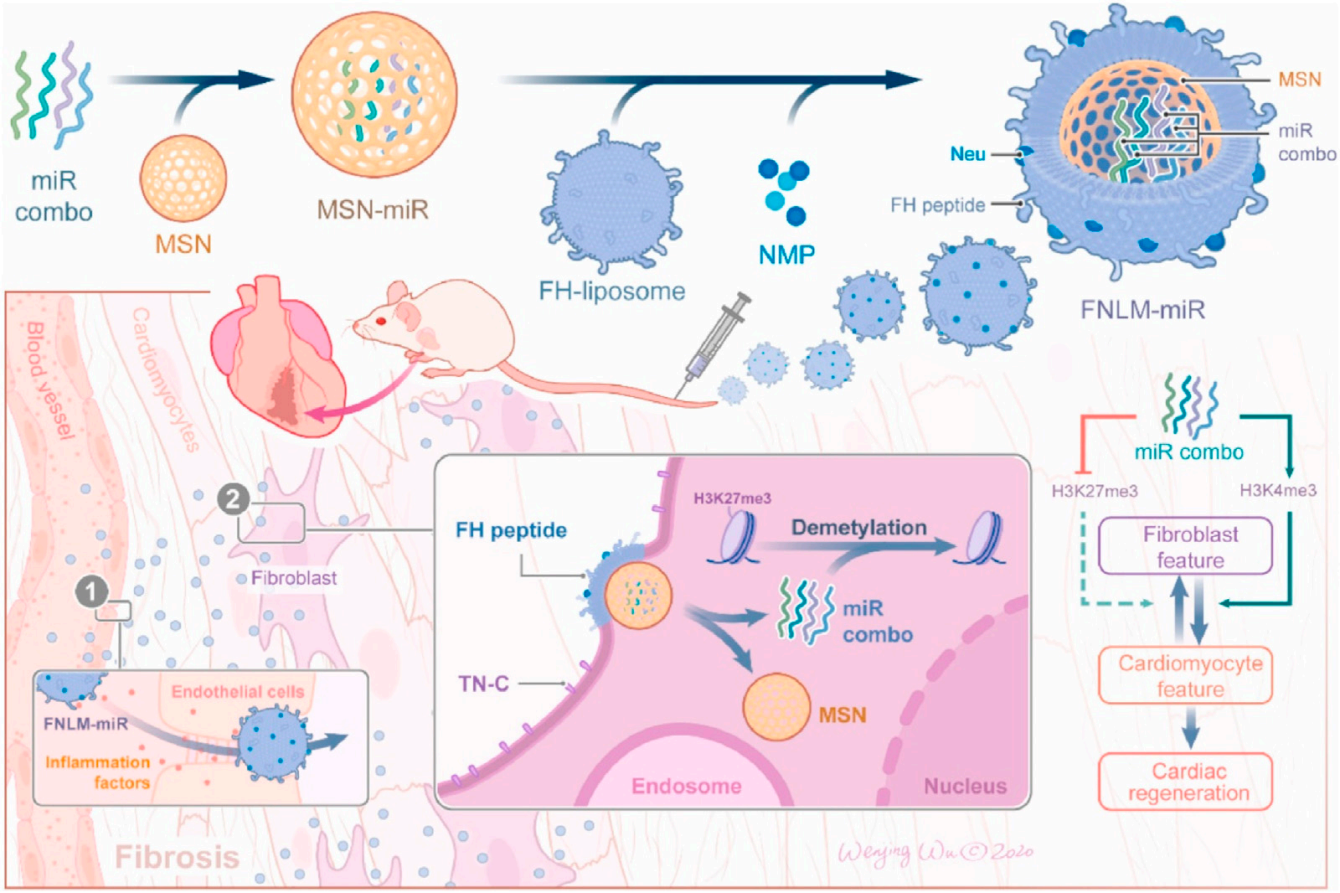
Schematic diagram of the preparation process of FNLM miR and application in myocardial ischemia/reperfusion injury model (Wang et al., 2021).

Porous silicon nanoparticles (MSV) have good biocompatibility and degradability and also possess unique functions, including high porosity (up to 80%) and a unique chemical surface that can enhance the solubility of hydrophobic drugs and control drug release. These properties make MSVs highly suitable for the delivery of therapeutic agents, potentially improving treatment outcomes and reducing side effects (Wang et al., 2023; Petrisor et al., 2022). Ma et al. (Ma et al., 2016) specifically coupled e-selectin with thioaptamer (ESTA) and covalently connected it to the surface of MSV, successfully designing a nanoparticle that can target atherosclerotic inflammatory endothelial cells (ESTA-MSV). MiR-146a and miR-181b were compressed in ESTA-MSV nanoparticles and injected into ApoE gene-deficient mice through the tail vein. The findings indicated that ESTA-MSV-encapsulated mir-1461/181b could effectively suppress the expression of chemokines in aortic tissue, improve endothelial cell inflammation and significantly shorten the thickness of atherosclerotic plaques. Mesoporous silica (MSN) possesses the advantages of a large and uniform pore structure, high specific surface area, an adjustable pore structure, excellent chemical stability, and versatility, which makes it a crucial material in adsorbents, catalysts, separation materials, and drug-controlled release systems. Wang et al. (Wang et al., 2021) developed a novel biomimetic non-viral vector by wrapping the FH peptide-modified neutrophil membrane around MSNs loaded with miR-1, 133, 208, and 499 (miRCombo) (Figure 5). They injected MSNs-miR into the tail vein of a mouse model of myocardial ischemia/reperfusion injury, delivering miRCombo specifically to damaged cardiac fibroblasts (CFs) through the natural homing ability of neutrophil membrane protein and the high affinity of FH peptide to CFs. By mediating microRNA regulation, they transformed CFs into induced cardiomyocyte-like cells (iCMs), achieving *in vivo* cardiac reprogramming and improving heart function while reducing fibrosis. Cheang et al.’s (Cheang et al., 2012) research team used aminopropyltriethoxysilane (APTES), a common chemical, to covalently bind to the silicon atoms on the surface. By modifying the surface, APTES enhanced the binding ability to plasmid DNA. This novel gene carrier preparation improved the delivery efficiency of plasmid DNA in human vascular smooth muscle cells.

Despite the promise of inorganic nanoparticles for delivery, diagnostic, and therapeutic applications, the *in vivo* degradability and toxicity of inorganic nanoparticles compared to organic nanoparticles have long been an issue of concern. After intravenous injection, inorganic nanoparticles may selectively aggregate and accumulate in specific tissues or organs, and long-term deposition is often toxic, thus limiting their clinical applications (Yang et al., 2020). Accurately comparing the toxicity of inorganic nanoparticles is often challenging due to the variation in dose levels, routes, purity, and frequency of administration reported in various studies (Mohammadpour et al., 2019). Typically, inflammation and oxidative stress are common mechanisms for inducing toxicity in inorganic nanomaterials (Cheng et al., 2022). For example, iron oxide nanoparticles increase endothelial barrier permeability by inducing oxidative stress, which can lead to local inflammation (Apopa, 2009); Hsu et al. (Hsu et al., 2021) reported that exposure to silicon oxide nanoparticles lead to a significant upregulation of TNF and MAPK signaling pathways. This upregulation triggered the secretion of various pro-inflammatory cytokines by modulating the p38 and JNK signaling pathways, ultimately activating the transcription factor AP-1. In turn, AP-1 promotes apoptosis and inflammatory responses. The main molecular mechanism underlying the toxicity of gold nanoparticles is the increase in oxidative stress caused by the formation of free radicals. This leads to oxidation and damage to intracellular components, lipids, proteins, and DNA. Additionally, the aggregation of nanoparticles following systemic administration not only results in a loss of function but may also cause capillary occlusion, resulting in end-organ damage (Lanone and Boczkowski, 2006; Aillon, 2009; Thakor et al., 2011). Therefore, in the future use of inorganic nanoparticles for drug delivery and therapeutic applications, it is not only necessary to emphasize their success in applications, but also to pay attention to the toxicity of inorganic nanoparticles due to their non-degradability, and to conduct a comprehensive assessment and research to minimize the potential toxicity and side effects.

In summary, this is a review of several commonly used non-viral vectors in gene therapy of cardiovascular diseases in recent years, and the specific summary can be found in Tables 2, 3.

**TABLE 2:** To summarize the advantages and disadvantages of non-viral vectors.

		Advantages	Disadvantages
Polymer	PEI	a. High transfection efficiency	a. Highly cytotoxic
		b. Good intracellular release performance	b. Non-degradability
		c. Adjustable molecular weight and chemical structure	c. Low stability
	PAMPM	a. High transfection efficiency	a. Non-degradability
		b. Adjustable molecular weight and chemical structure	b. Highly cytotoxic
	Chitosan	a. Biodegradability	a. Low transfection efficiency
		b. High biocompatibility	b. Spontaneous aggregation
			c. Susceptible to environmental influences
	PLGA	a. Biodegradability	a. Complex synthesis methods
		b. High biocompatibility	b. The rate of degradation was not controllable
Liposome		a. Biocompatibility	a. Poor stability in the body
		b. Amphiphilic drug loading	b. Potential side effects
		c. Targeting effect	
LNP		a. High transfection efficiency	a. Limited load capacity
		b. Easy to surface modification	b. Potential side effects
		c. Applicable to small molecular nucleic acid delivery	c. High production cost
	AuNPs	a. Small size	a. High production cost
		b. Easy to surface modification	b. Non-degradability
Inorganic Nanoparticles	MNP	a. Easy to surface modification	c. Non-degradability
		b. Superparamagnetism	d. Potential side effect
	MSV	a. Easy to surface	a. Complex synthesis methods
		b. Biodegradability	b. High production cost
		c. High biocompatibility	
	MSN	a. Controllable pore structure	a. Potential side effects
		b. Chemical stability	b. Synthetic methods are complex and costly
		c. Easy to surface	

**TABLE 3:** To summarize the application strategies of non-viral vectors in the delivery of cardiovascular diseases.

	Mode of delivery	Strategies for targeting	Type of nucleic acid delivered	References
Polymer	Intravenous or subcutaneous injection	Polypeptide, Anti-e-selectin mab (1.2B6), Short peptide (Arg-Glu-Asp-Val), Galactose, Anti-tie2 +Tie1 antibodies, Cell membrane	siRNA, miRNA	Theoharis et al. (2009), Moon et al. (2014), Wang et al. (2016), Zhou et al. (2016), Jiang et al. (2022), Messerschmidt et al. (2022), Wang et al. (2022)
Liposome	Intravenous or subcutaneous injection	Cardiac myosin 2G4 mAb, Anti-VCAM-1-Fab’ mAb、Anti-P-selectin, Anti-ICAM-1 mAb, transferrin	miRNA, siRNA, plasmid	Danila et al. (2009), Ko et al. (2009), Scott et al. (2009), Kang et al. (2011)
LNP	Intravenous or subcutaneous injection	Anti-CD5, Mannose, Cell membrane	mRNA, siRNA, plasmid	Rurik et al. (2022), Gao et al. (2023), Huang et al. (2023)
Inorganic Nanoparticles	Intravenous or local injection	Pigallocatechin-3-gallate (EGCg)、Anti-E-selectin、Polypeptide (CREKA)、	siRNA, plasmid	Song et al. (2014), Tarin et al. (2015), Ma et al. (2016), Wang et al. (2021)
		Anti-CD163, Thioaptamer (ESTA), Anti-CD36, Cell membrane, aminopropyltriethoxysilane (APTES)		

## 3 Summary and outlook

Over the past decade, nanotechnology has advanced rapidly. Nanotechnology-mediated gene delivery provides a broader view of CVD gene therapy. In this paper, we reviewed the application of common non-viral nanocarries (polymers, liposomes, and inorganic nanoparticles) in CVD gene therapy. With superiority in half-life extension and biocompatibility, non-viral nanocarriers actively deliver exogenous nucleic acids to target cells or organs, marking the dawn of a new era for treating CVD. Although non-viral nanocarries have great application prospects and satisfactory preclinical results, nanotechnology-mediated non-viral CVD gene therapy has not been successfully applied clinically. Clinical translation of nanomedicine still has a long way to go. Multiple reasons account for this defeat: 1) inadequate gene delivery to target sites thus destroying the potential of transgenes. 2) insufficiency of transgenes expression time. 3) lack the recognition of underlying pathophysiological mechanisms. In addition, the scale-up of non-viral vectors is a challenge, and there is a need to develop scale-up methods that ensure consistency, reproducibility, and product quality and safety of the final product. In addition, non-viral vectors are usually made of synthetic polymers or lipids, which may not be readily degradable, leading to toxicity, an immune response, and long-term accumulation of the vector in the body. Therefore, there is a need to develop non-viral vectors that are biodegradable and have minimal toxic effects for clinical applications. Clinical regulation also plays an important role in the development of non-viral vectors for use in the field of CVD gene delivery. Regulatory agencies such as the U.S. Food and Drug Administration (FDA) require rigorous safety and efficacy evaluations of gene therapy products before they can be approved. This includes preclinical testing in animal models to assess the safety and efficacy of the product and clinical trials in humans to assess its safety and efficacy. Regulatory requirements for non-viral vectors may vary depending on the country or region in which they are used, and investigators need to ensure that local regulatory requirements are met. As nanomedicine advances, continuous improvement and optimization of nano-gene delivery vectors will help overcome these obstacles. In addition, combination therapy like gene-surgery therapy is promising to provide a comprehensive strategy for CVD treatment. More and more optimized and modified nanoscale gene delivery systems are believed to enter and successfully applied in clinics in the near future.
